# Self-cleaning MOF: realization of extreme water repellence in coordination driven self-assembled nanostructures†
†Electronic supplementary information (ESI) available: Experimental details and characterization. See DOI: 10.1039/c5sc03676c


**DOI:** 10.1039/c5sc03676c

**Published:** 2015-12-11

**Authors:** Syamantak Roy, Venkata M. Suresh, Tapas Kumar Maji

**Affiliations:** a Molecular Materials Laboratory , Chemistry and Physics of Materials Unit , Jawaharlal Nehru Centre for Advanced Scientific Research , Jakkur , Bangalore-560064 , India . Email: tmaji@jncasr.ac.in

## Abstract

Bio-inspired self-cleaning surfaces have found industrial applications in oil–water separation, stain resistant textiles, anti-biofouling paints in ships *etc.* Interestingly, self-cleaning metal–organic framework (MOF) materials having high water contact angles and corrosion resistance have not been realized so far. To address this issue, we have used the fundamentals of self-assembly to expose hydrophobic alkyl chains on a MOF surface. This decreases the surface free energy and hence increases hydrophobicity. Coordination directed self-assembly of dialkoxyoctadecyl-oligo-(*p*-phenyleneethynylene)dicarboxylate (**OPE-C_18_**) with Zn^II^ in a DMF/H_2_O mixture leads to a three dimensional supramolecular porous framework {Zn(OPE-C_18_)·2H_2_O} (**NMOF-1**) with nanobelt morphology. Inherently superhydrophobic and self-cleaning **NMOF-1** has high thermal and chemical stability. The periodic arrangement of 1D Zn-OPE-C_18_ chains with octadecyl alkyl chains projecting outward reduces the surface free energy leading to superhydrophobicity in **NMOF-1** (contact angle: 160–162°). The hierarchical surface structure thus generated, enables **NMOF-1** to mimic the lotus leaf in its self-cleaning property with an unprecedented tilt angle of 2°. Additionally, superhydrophobicity remains intact over a wide pH range (1–9) and under high ionic concentrations. We believe that such a development in this field will herald a new class of materials capable of water repellent applications.

## Abstract 2

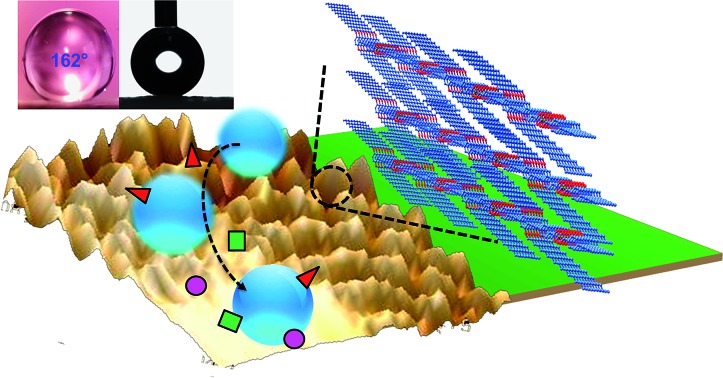
A superhydrophobic self-cleaning MOF nanostructure has been synthesized using a unique ligand design strategy and coordination directed self-assembly. The material has hierarchical surface roughness and is stable under extreme corrosive conditions.

## Introduction

The progress in scientific research has witnessed the efforts of scientists to mimic the intricate and precise design of nature for fabricating novel functional materials.^1^–^6^ In this context, the “Lotus Effect” has incurred immense interest for designing biomimetic self-cleaning materials.^7^–^10^ It involves easy rolling of water droplets over the lotus leaf. The leaf surface shows characteristic micro- and nanoscale roughness with a “re-entrant texture” which is determined to be a prerequisite for such water repellence.^11^–^15^ This multiscale or hierarchical roughness reduces the surface free energy, increases the static contact angle (CA) (>150°) and reduces the tilt angle (<10°) leading to superhydrophobic surfaces with self-cleaning properties.^16^–^18^ Therefore, the shape, size, rigidity and ordering with combined surface micro- and nanostructure are the guiding principles for the design of artificial self-cleaning materials.^19^–^22^ Towards this, carbon nanotubes (CNTs),^23^,^24^ lithographic patterning,^25^–^28^ aligned polymer nanofibers,^29^–^31^ self-assembled monolayer (SAM) modified surfaces^32^,^33^ have been successfully designed as superhydrophobic surfaces with very high CAs. In this context, superhydrophobic metal–organic frameworks (MOFs)^34^–^46^ would provide the advantage of both inherent porosity and surface water repellence. This could lead to applications of this material in oil–water separation membranes, waste water treatments and fuel purification technology.^47^–^50^

Recently, MOFs have been studied for superhydrophobic applications either *via* post-synthetic modification (PSM) by fluoroalkyl chains or by generating external surface corrugation.^51^–^58^ Kitagawa *et al.* proposed a novel method of synthesizing superhydrophobic MOF materials by generating external surface corrugation derived from aromatic surface groups.^52^ However, it lacked the basic requirements for practical applicability *i.e.* solution processability and self-cleaning properties. In this context, coordination driven self-assembly of a rigid π-conjugated organic linker containing long hydrophobic alkyl chains^59^,^60^ would provide solution processable nanoMOF (NMOF)^61^–^65^ structures. Here, the surface free energy will be dictated by the alkyl chains decorating the NMOF surface and create a low adhesion surface for water droplets. We therefore conjectured that coordination driven self-assembly of oligo-(*p*-phenyleneethynylene) dicarboxylate with alkoxyoctadecyl (C_18_) chains (**OPE-C_18_**)^66^,^67^ would generate a supramolecular NMOF structure which would be promising for superhydrophobic self-cleaning applications.

**Scheme 1:**
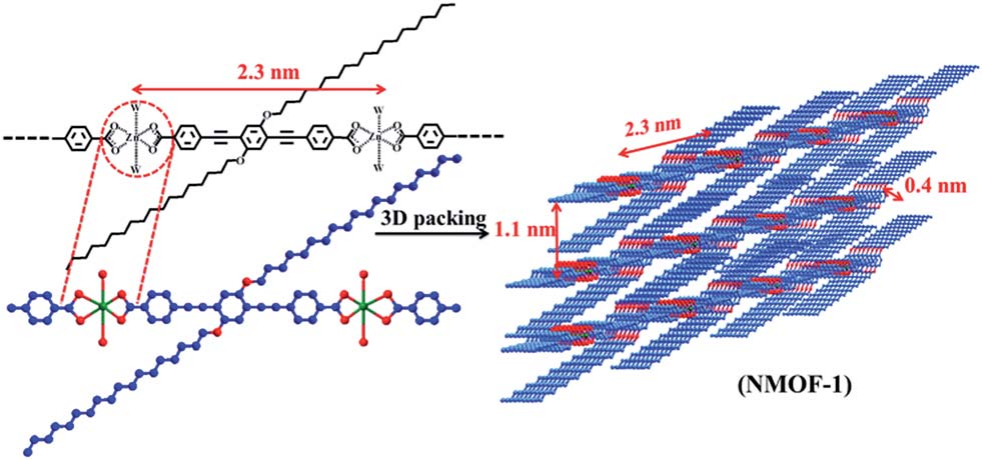
Bottom-up approach for the fabrication of a self-cleaning MOF nanostructure by coordination driven self-assembly between Zn^II^ and **OPE-C_18_**.

In this article, we report the rational design and synthesis of a self-cleaning nanoscale supramolecular 3D porous framework {Zn(OPE-C_18_)·2H_2_O} (**NMOF-1**) with inherent superhydrophobicity based on the self-assembly of **OPE-C_18_** and Zn^II^ (Scheme 1). The micro-spaces between **NMOF-1** micro-particles are capable of creating a solid–air–water composite interface to develop high moisture resistance. The nanoscale roughness is created by the presence of periodic alkyl chains that generate a hierarchical structure with pH tolerability. Our design is an easy and straightforward bottom-up approach for the construction of superhydrophobic MOF nanostructures with remarkably low sliding angles and excellent self-cleaning properties extendable to large areas. To the best of our knowledge, the fabrication of self-cleaning MOFs and an in-depth analysis of their characteristic surface structure are yet to be reported.

## Results and discussion

**Fig. 1:**
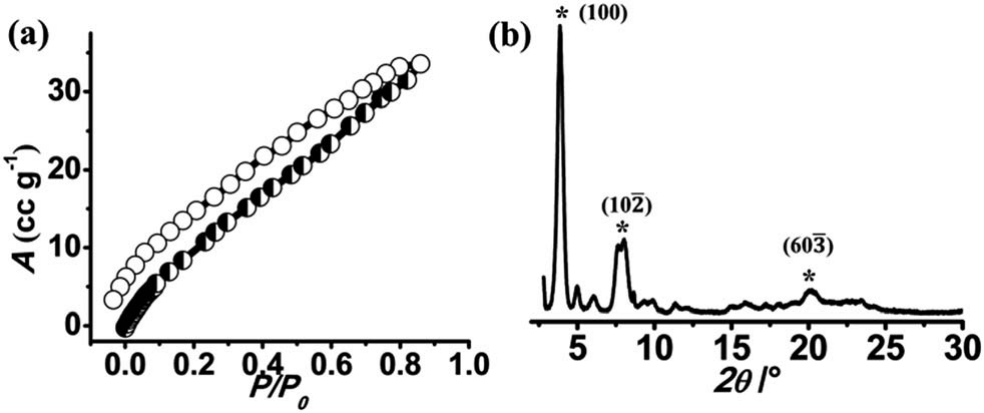
(a) CO_2_ (at 195 K) adsorption isotherm of de-solvated **NMOF-1**. (b) PXRD pattern of **NMOF-1**.

**NMOF-1** has been synthesized by stirring **H_2_OPE-C_18_** and Zn(OAc)_2_·2H_2_O in a DMF/H_2_O mixed solvent system in the presence of triethylamine at room temperature.^68^ Coordination of Zn^II^ to **OPE-C_18_** is evidenced by the spontaneous formation of colloidal turbidity on addition of the aqueous Zn(OAc)_2_ solution. The reaction mixture is stirred for 2 hours at room temperature and centrifuged. EDAX analysis of the resulting colloid showed the presence of Zn^II^ (Fig. S1†) and CHN analysis suggests a molecular formula of {Zn(OPE-C_18_)·2H_2_O} (**NMOF-1**), thereby proposing 1 : 1 coordination of Zn^II^ ions with **OPE-C_18_**. FT-IR spectrum of **NMOF-1** shows strong peaks at 1595 cm^–1^ and 1413 cm^–1^, corresponding to asymmetric and symmetric stretching vibrations of carboxylate groups and the difference (*Δ* value) is found to be 182 cm^–1^ (Fig. S2†). This suggests a bidentate coordination mode of the Zn^II^ metal ion to **OPE-C_18_** (Scheme 1). The presence of a broad peak at 3460 cm^–1^ confirms the presence of Zn^II^ bound coordinated water molecules. These results suggest a hexacoordination environment around Zn^II^ ion with four oxygens from two carboxylate groups of **OPE-C_18_** and two coordinated water molecules. Thermogravimetry analysis further indicates its stability up to 340 °C (Fig. S3†). The N_2_ adsorption isotherm at 77 K of **NMOF-1** shows a type II uptake profile (Fig. S4†). However, the CO_2_ adsorption isotherm at 195 K showed a gradual uptake of CO_2_ with increasing pressure (Fig. 1a). The final amount is observed to be 35 cm^3^ g^–1^ at *p* = 1 atm which corresponds to 1 molecule of CO_2_ per formula unit of **NMOF-1**. The permanent porosity indicates the presence of a porous 3D structural organization in **NMOF-1** (Scheme 1). Powder X-ray diffraction (PXRD) suggests high crystallinity of **NMOF-1** (Fig. 1b). Indexing of the PXRD pattern provided insights into its packing. Modelling through the Crysfire software^68^ suggests a monoclinic crystal system of **NMOF-1** with a cell volume of 2197 Å^3^ and cell parameters of *a* = 29.40(5) Å, *b* = 4.146(7) Å and *c* = 22.81(5) Å. The close equivalence of the experimental and indexing results indicates the accuracy of the calculation (Table S1†). The (100) peak at 3.9° (*d* = 23 Å) corresponds to the repeating distance of Zn^II^ centres connected by **OPE-C_18_**, suggesting the formation of a 1D coordination chain. Therefore the coordination of the terminal carboxylate groups of **OPE-C_18_** to Zn^II^ extends **NMOF-1** in 1D (Scheme 1). The diffraction peak (603). The diffraction peak (603̄) at 20.2° () at 20.2° (*d* = 4.4 Å) indicates weak π–π interactions between the **OPE-C_18_** unit of 1D chains that result in 2D layers. Such stacking of the 2D layers is reinforced by the orientation of adjacent alkyl chains indicated by the (102 unit of 1D chains that result in 2D layers. Such stacking of the 2D layers is reinforced by the orientation of adjacent alkyl chains indicated by the (102̄) peak at 8° () peak at 8° (*d* = 11 Å). The long alkyl chains interact *via* van der Waals forces to extend the packing into a 3D supramolecular framework. This generates a supramolecular porous structure extended *via* lamellar packing between adjacent **OPE-C_18_** units of two successive **NMOF-1** layers (Scheme 1). Interestingly, **NMOF-1** retained its structural integrity even at higher temperatures as evident from temperature dependant PXRD measurements (Fig. S5†). Measurements were carried out at three different temperatures; PXRD analysis at 100 °C, 200 °C and 300 °C showed that the characteristic peaks of **NMOF-1** are retained at all temperatures. This result clearly reveals the exceptional thermal stability of **NMOF-1**.

### Morphology transformation

**Fig. 2:**
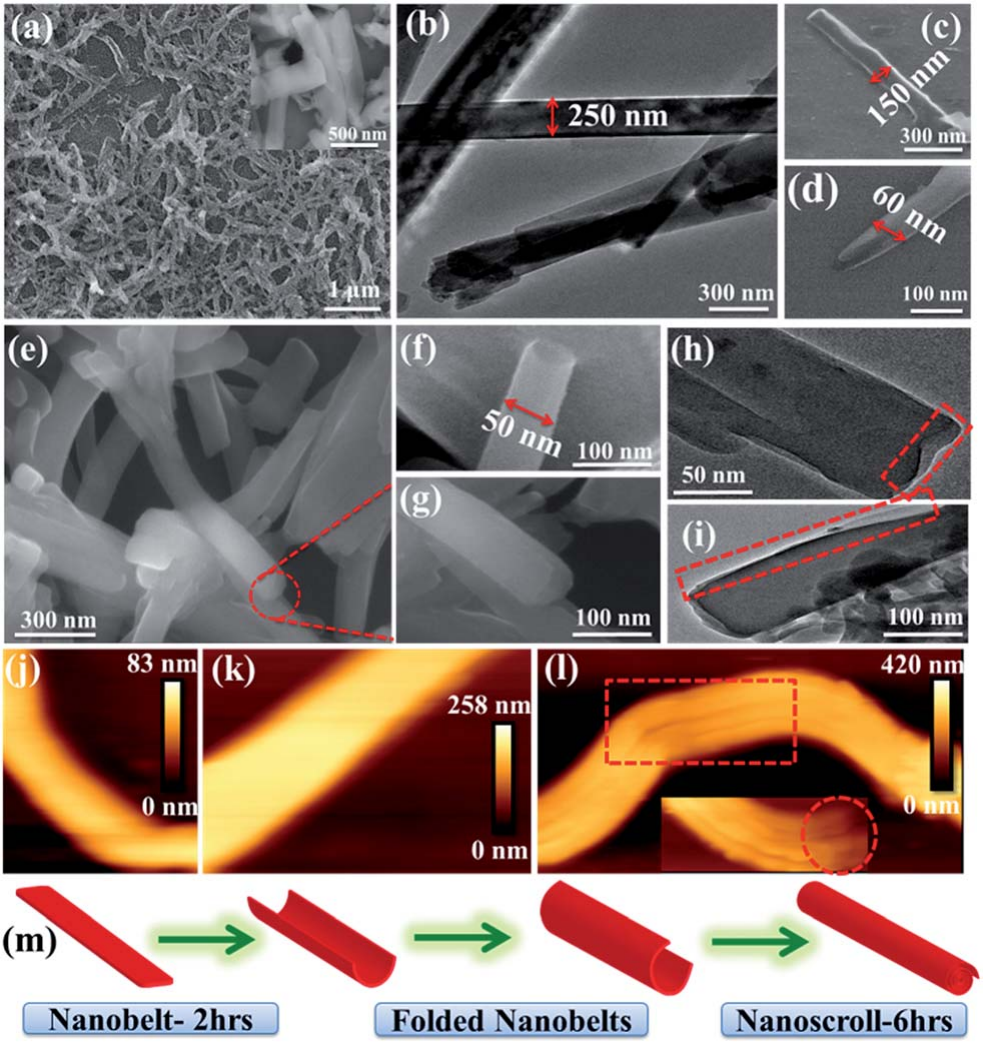
(a) FESEM image (inset: nanobelts at higher magnification) and (b) TEM image of **NMOF-1** nanobelts. (c and d) FESEM images of semi-scrolled nanobelts. (e–g) FESEM and (h and i) TEM images of nanoscrolls at different magnifications showing the changes in cross section upon scrolling. AFM image of (j and k) nanobelt and (l) nanoscroll (inset: single nanoscroll showing opening at the mouth). (m) Schematic showing the possible morphological transformation of nanobelts to nanoscrolls in **NMOF-1**.

The nanomorphology of **NMOF-1** was investigated using field emission scanning electron microscopy (FESEM), transmission electron microscopy (TEM) and atomic force microscopy (AFM). FESEM, TEM and AFM revealed the presence of belt-like nanostructures of **NMOF-1** (Fig. 2a, b, j, k and S6†). The nanobelts offered a length of 700–1000 nm and a width of 200–300 nm. The height profile analysed using AFM was found to be about 80 nm and as shown in Scheme S2,† the length of the **NMOF-1** nanobelts is composed of the 1D chains of Zn-OPE-C_18_. The width of the belts is formed by the weak π–π interactions between the 1D chains forming the 2D layer of the nanobelts. Finally the height of the nanobelts is formed by the 3D packing of **NMOF-1***via* the interaction of the alkyl chains. Interestingly, when the reaction time was increased to 6 hours, nanoscrolls of **NMOF-1** were observed. FESEM and TEM measurements further confirmed the formation of the nanoscrolls from the nanobelts (Fig. 2e–i and S7†). Intermediate reaction times offered semi-scrolled nanostructures having a reduced width (Fig. 2c and d and S8†). Upon complete transformation the nanoscrolls showed a length of 400–800 nm and a width of 40–100 nm. The AFM height profile of the nanoscrolls showed a value of about 270 nm (Fig. S9†). The increase in height of the nanoscrolls is attributed to the scrolling effect. These results suggest the scrolling of the belt occurs in a longitudinal direction leaving long alkyl chains at the surface of the nanobelts and nanoscrolls. As the reaction progresses, the belts start to scroll up to minimize the repulsive forces between the alkyl chains of **OPE-C_18_** and solvents in the reaction medium (Fig. 2m, Scheme S2†). To study the effect of dynamicity of the alkyl chains on the surface, nanoscrolls of **NMOF-1** were allowed to stand in acetonitrile, a less polar solvent than water. After 4 days of standing in solution, partially unscrolled structures of **NMOF-1** taking the shape of nano-containers were observed using FESEM analysis (Fig. S10†). A decrease in the hydrophobic interactions between the alkyl chains in a less polar solvent allows the scrolls to relax and open up along the long-alkyl chain direction therefore resulting in a new morphology of **NMOF-1**.

### Superhydrophobicity and surface analysis

**Fig. 3:**
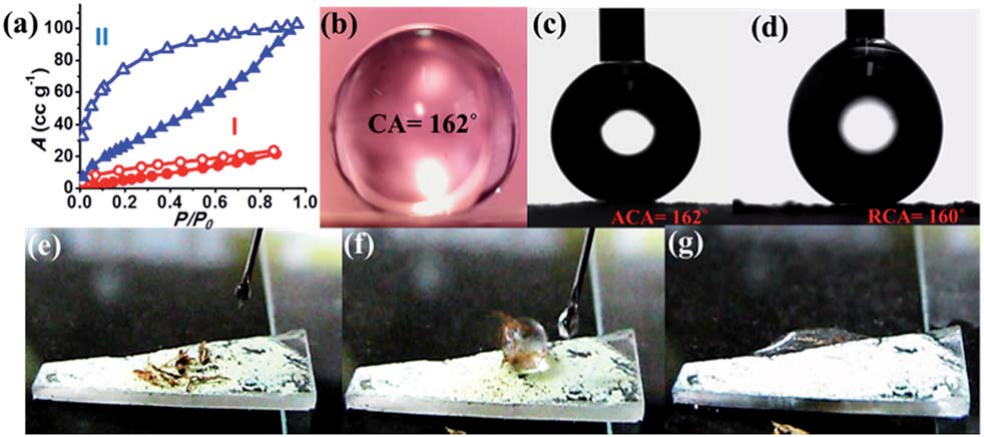
(a) Solvent vapor adsorption isotherms of **NMOF-1** at 298 K: (I) water (red) and (II) benzene (blue), *P*_0_ is the saturated vapour pressure; 3.17 kPa (water) and 12.60 kPa (benzene) at 298 K. Water contact angles of **NMOF-1** coated substrate: (b) static contact angle, (c) advancing contact angle and (d) receding contact angle (e–g). Video snapshots of self-cleaning experiment showing the removal of dirt from surface.

The presence of alkyl chains and the dynamicity along the surface and pores of the nanostructures further motivated us to study the hydrophobic properties of **NMOF-1**. To investigate the polarity of the self-assembly and supramolecular organization, we carried out water and benzene adsorption experiments at room temperature. The water adsorption isotherms of **NMOF-1** showed negligible uptake in the low pressure region with gradual uptake at higher pressures, finally reaching 20 mL at *P*/*P*_0_ = 1.0. This suggests the hydrophobic nature of the framework (Fig. 3a). The hydrophobicity of the pore surface was further validated by the benzene adsorption isotherms showing an uptake of 102 cm^3^ g^–1^ (Fig. 3a) which corresponds to ∼1 benzene molecule per formula unit of **NMOF-1**. Therefore, its pore can be utilized for polar/non-polar solvent separations.

**Fig. 4:**
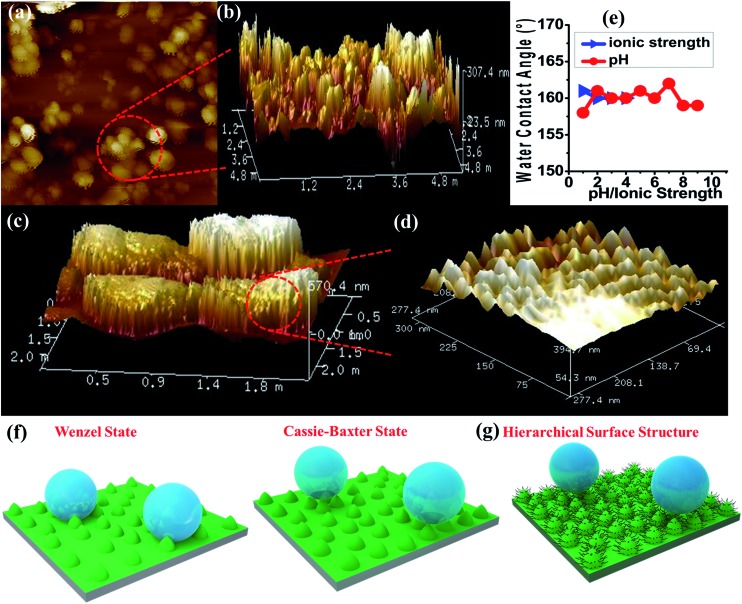
(a) 2D-AFM image of **NMOF-1** micro-particles on a coated glass surface and (b) corresponding 3D-AFM image. (c) 3D-AFM image of four adjacent microspheres of **NMOF-1** and (d) corresponding high magnification image showing continuous nano-roughness present on each micro-particle. (e) Plots showing changes in CA with pH/ionic strength. (f) Schematic diagrams showing the different states used to explain surface water repellence: left: Wenzel state or the wetting state. Right: Cassie–Baxter state or the superhydrophobic state (a transition from the Wenzel to the Cassie–Baxter model occurs when we consider that rough textures on a surface trap air-pockets in between) and (g) incorporation of hierarchical surface for the generation of the self-cleaning effect in **NMOF-1**.

For surface characterization, the free organic linker (**H_2_OPE-C_18_**) was initially investigated for superhydrophobicity. The contact angle was determined to be 140–147°, proving the hydrophobicity of **H_2_OPE-C_18_** (Fig. S11†). However, superhydrophobicity was not realized. Hydrophobicity of the **NMOF-1** surface was then investigated by coating its ethanolic dispersion on a glass substrate. The nanoscale structure of **NMOF-1** makes it highly solution processable and easy to coat on glass. Water contact angles were measured on **NMOF-1** coated glass substrates and were determined to be 160–162° in a circle fitting mode (Fig. 3b). These high contact angle values are the highest reported for MOFs without any post-synthetic modifications of the pore as well as the external surface (Table 1). Therefore, to gain insight into this superhydrophobic property of **NMOF-1**, FESEM and AFM analysis was performed. FESEM images of coated **NMOF-1** shows a uniform distribution of spherical particles with sizes in the range 10–30 μm (Fig. S12†). The occurrence of such particles generates the micro-roughness with trapped air pockets in between them. This distribution is ideal for hydrophobic applications. To investigate further, non-contact mode AFM imaging of **NMOF-1** was performed. 2D AFM images authenticated the presence of spherical particles of **NMOF-1** (Fig. 4a). The particles show needle like protrusions in the nanoscale regime. The 3D AFM image shows the height of the spheres in the range of 300–500 nm (Fig. 4b). The 3D AFM image of a single sphere validates the presence of spikes 50–100 nm wide with an inter-spacing of 10–50 nm having a height of 200–300 nm (Fig. 4c and d). These descriptions confirm the presence of hierarchical surface roughness. Such periodic existence of large and small particles generates areas containing peaks and troughs throughout the surface (Fig. 4b and d). This is commonly known as the “hills and valley” type surface terrain with trapped air pockets in between. The hills and valley are observed even in the nanoscale regime. The Cassie–Baxter model^69^,^70^ (Fig. 4f) predicts that such surface roughness will lead to a superhydrophobic structure. The uniformity in micro/sub-microscale roughness of **NMOF-1** therefore provides an ideal surface for super-hydrophobic and self-cleaning applications.

**Table 1:** Reported superhydrophobic MOFs in the literature highlighting the comparison of contact angles, stability in the pH range and self-cleaning properties with the current work

MOF	CA (°)	PSM	Self-cleaning	pH stability range	Reference
MOFF-2	151 ± 1	No	—	—	53
MOFF-3	135 ± 2	No	—	—	53
MIL-53(Al)-AM4	>150	Yes	—	—	51
MIL-53(Al)-AM6	>150	Yes	—	—	51
NH_2_-MIL-53(Al)	151–169	Yes	—	—	55
PESD-1	>150	No	—	—	52
**NMOF-1**	160–162	No	Yes	1–9	Current work

### Self-cleaning properties

The advancing and receding contact angles of **NMOF-1** were measured to be 162° and 160° respectively, leading to a very small contact angle hysteresis of 2° (Fig. 3c and d). This further prompted us to investigate its self-cleaning properties (Fig. S13†). This was examined by placing dust particles on the **NMOF-1** coated glass surface. The water droplets indeed rolled off the surface carrying the dust particles along with it (Fig. 3e–g). The whole experiment was video recorded (Video S1 and S2†). However, for any practical applications of **NMOF-1**, it must be stable under a variety of extreme conditions such as high acidic and basic conditions and also solutions of high ionic strength. An experiment was designed where aqueous solutions of different pH were used to measure contact angles. Interestingly, the glass surface with a **NMOF-1** coating showed amazing stability with high contact angles in the entire acidic pH range (1–6) and also under mildly basic conditions (up to pH = 9). The contact angle varied from its original value only slightly in the entire range (pH = 1 to 9). Also under high ionic strength solutions, contact angles showed only a minimal change (*I* = 1–4) (Fig. 4e and S14†).

The supramolecular packing of **NMOF-1** leaves the alkyl chains of **OPE-C_18_** both between the 2D layers as well as on the external surface (Scheme S2†). On coating the glass surface, spherical organizations of **NMOF-1** nanobelts were observed which create air pockets in between two successive particles. This prevents the wetting of the surface. Also the periodic alignment of alkyl chains on the surface creates nano-spaced roughness generating a hierarchical surface structure which assists the easy rolling of water droplets on it (Fig. 4g, Video S1 and S2†). Likewise, the presence of long alkyl chains on the surface of **NMOF-1** shields the framework from decomposition under such extreme conditions rendering it a highly stable self-cleaning material. The PXRD peaks showed good correspondence before and after the self-cleaning experiment (Fig. S15†) indicating the robustness of **NMOF-1** in such applications. The results were compared to other reported MOFs and have been tabulated in Table 1.

## Conclusions

In conclusion, a novel ligand design strategy has been exploited for the fabrication of an inherently superhydrophobic and self-cleaning nanoscale metal–organic framework **NMOF-1**. The control of reaction parameters has generated nanobelts and nanoscroll morphologies. The micro/nanoscale surface roughness generated was also cultivated for the superhydrophobic and self-cleaning application of **NMOF-1** which is unique in MOF chemistry. Its exceptional stability under extreme acidic, basic and ionic conditions renders its applicability as a pH stable self-cleaning and luminescent material. The shape-shifting porous hybrids can also be utilized for a host of dimension dependent applications like optical waveguiding, charge transport *etc.* Exploitation of the supramolecular organization and nanomorphology of MOFs for large area coating and its in-depth surface analysis is done for the first time in this work. This could potentially open up new avenues in the design of superhydrophobic self-cleaning MOF materials without tedious post-synthetic modifications and usher in a new class of materials meeting industrial needs.

## Supplementary Material

Supplementary information

Supplementary movie

Supplementary movie
