# Supplementary material for: Self-cleaning MOF: realization of extreme water repellence in coordination driven self-assembled nanostructures
Source: Chem Sci. 2015 Dec 11;7(3):2251–6. doi: 10.1039/c5sc03676c (PMC5977372; doi:10.1039/c5sc03676c)
Supplement: Supplementary file 1 [file SC-007-C5SC03676C-s001.pdf]

Electronic Supplementary Material (ESI) for Chemical Science.
This journal is © The Royal Society of Chemistry 2015

Self-cleaning MOF: Realization of Extreme Water Repellence in Coordination Driven Self-Assembled Nanostructures

Syamantak Roy,[a] Venkata M. Suresh,[a] and Tapas Kumar Maji[a]*

a Molecular Materials Laboratory, Chemistry & Physics of Materials Unit (CPMU), Jawaharlal Nehru Centre for Advanced Scientific Research, Jakkur, Bangalore-560064, India

* E-mail: tmaji@jncasr.ac.in, Phone: +91-802208-2932

Supporting Information

1. Experimental Section……………………………………………………………….2
2. Synthetic Procedure……………………………………………………………...4
3. EDAX Spectrum………………………………………………………………….5
4. FT-IR Spectra…………………………………………………………………….5
5. TG Analysis……………………………………………………………………...6
6. N_2 Adsorption Profile……………………………………………………………6
7. PXRD analysis……………………………………………………………………..7
8. FESEM images of **NMOF-1** nanobelts……………………………………………9
9. FESEM images of **NMOF-1** nanoscrolls…………………………………………10
10. FESEM images of semiscrolled **NMOF-1**………………………………………11
11. AFM Height Profile Analysis……………………………………………………12
12. Structure-nanomorhology correlation of **NMOF-1**……………………………...13
13. FESEM analysis of reversibility of morphology transformation………………………14
14. Water contact angles of **H_2OPEC_{18}** coated glass surface………………………………..15
15. Superhydrophobicity models………………………………………………………15

16. FESEM analysis of **NMOF-1** coated glass substrate…………………………………....16

17. Video Snapshots of self-cleaning experiment………………………………………..16

18. Water contact angles under extreme conditions of **NMOF-1** coated glass substrate…...17

19. PXRD analysis before and after self-cleaning experiments……………………………..17

20. References……………………………………………………………………………18

1. Experimental section

Materials

$Pd(PPh_3)_4$ and $Zn(OAc)_2 \cdot 2H_2O$ were obtained from Sigma-Aldrich Chemical Co and cuprous iodide was obtained from Loba Chemie Pvt. Ltd. N,N-dimethyl formamide (DMF) and tetrahydrofuran (THF) were obtained from Spectrochem Pvt. Ltd (Mumbai, India). Tetrahydrofuran was pre-dried using standard procedure and all other reagents, solvents were of reagent grade and used without further purification.

Physical Measurements

Infrared spectral studies were done by making samples with KBr pellets using Bruker FT-IR spectrometer. Thermal stability of the **NMOF-1** is analyzed using Mettler Toledo TGA 850 instrument under inert atmosphere in the temperature range of 25-1000 °C at a heating rate of 3 °C /min. The elemental analyses were carried out using a Thermo Scientific Flash 2000 CHN analyzer. Powder X-ray diffraction studies were recorded on a Bruker D8 discover instrument using Cu-Kα radiation. Morphological studies have been carried out using Lica-S440I Field Emission Scanning Electron Microscope (FESEM) by placing samples on a silicon wafer under high vacuum with an accelerating voltage of 10 kV. Transmission Electron Microscopy (TEM)

analysis has been performed using JEOL JEM-3010 with an accelerating voltage at 300 kV. For this analysis **NMOF-1** was dispersed in ethanol by sonication before drop casting on a carbon-coated copper grid. Energy dispersive spectroscopy (EDS) analysis was performed with an EDAX genesis instrument attached to the FESEM column. Height profiles of the nanostructures were acquired with a JPKSPM Data Processing software. UV–vis spectra were recorded on a Perkin Elmer Model Lambda 900 spectrophotometer. Fluorescence studies were accomplished using Perkin Elmer Ls 55 Luminescence spectrometer. Fluorescence confocal microscopy images were obtained from LSM 510 META-Carl Zeiss. The LSM image examiner was utilized for processing the images.

Adsorption Measurements

Porosity measurements were carried out using QUNATACHROME QUADRASORD-SI analyser at 77 K for N_2 and 195 K for CO_2. In the sample tube the adsorbent samples (~100-150 mg) were placed which had been prepared at 170 °C under a 1×10^{-1} Pa vacuum for about 12 h prior to measurement of the isotherms. Helium gas (99.999% purity) at a certain pressure was introduced in the gas chamber and allowed to diffuse into the sample chamber by opening the valve. The amount of gas adsorbed was calculated from the pressure difference (P_{cal} - P_e), where P_{cal} is the calculated pressure with no gas adsorption and P_e is the observed equilibrium pressure. All the operations were computer-controlled. Solvent vapor adsorptions were carried out at 298K using BELSORP AQUA 3 solvent vapor analyzer. A sample of about ~100 –150 mg was prepared by heating at 170 °C for about 12 h under vacuum (1×10^{-1} Pa) prior to measurement of the isotherms. The solvent molecules used to generate the vapor were degassed fully by repeated evacuation. Dead volume was measured with helium gas. The adsorbate was placed into the sample tube, then the change of the pressure was monitored and the degree of adsorption was

determined by the decrease in pressure at the equilibrium state. All operations were computer controlled and automatic.

Contact Angle Measurements

Contact angles were measured using an indigenous set up coupled with a Logitech camera for capturing the images. Contact angles were also measured using dedicated contact angle analyzer, OCA30 from Data Physics instrument (GmbH, Germany). 4 μL of the sessile water droplets were employed for measuring the static contact angles. A minimum of ten measurements were made.

2. Synthetic Procedure

Synthesis of NMOF-1

H_2OPE-C_{18} was synthesized according to reported Sonogashira Hagihara coupling procedure.[1] A mixture of **H_2OPE-C_{18}** (20 mg, 0.02 mmol) and $Zn(OAc)_2 \cdot 2H_2O$ (5 mg, 0.02 mmol) in a 1:1 DMF/H_2O mixture (10 mL) was stirred for 15 minutes. Then 120 μL triethylamine was added to the above reaction mixture and immediate appearance of a white colloidal turbidity was observed. After 2 hours, the reaction was stopped and the precipitate was centrifuged and washed well with THF and water to yield **NMOF-1** as a bright green powder. Elemental analysis: Calcd. for $C_{60}H_{86}O_8Zn$: C, 72.0; H, 8.7; Found: C,72.6; H, 8.5. FT-IR (cm^{-1}): 3430(s), 2913(s), 2851(s), 1690(s), 1595 (s), 1413(s), 1276(s), 1214(w), 860(w), 775(w), 550(w)

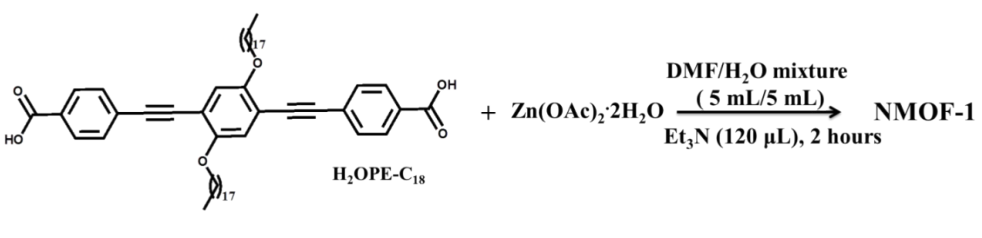

Scheme S1. Synthetic scheme for the fabrication of **NMOF-1.**

3. EDAX Spectrum

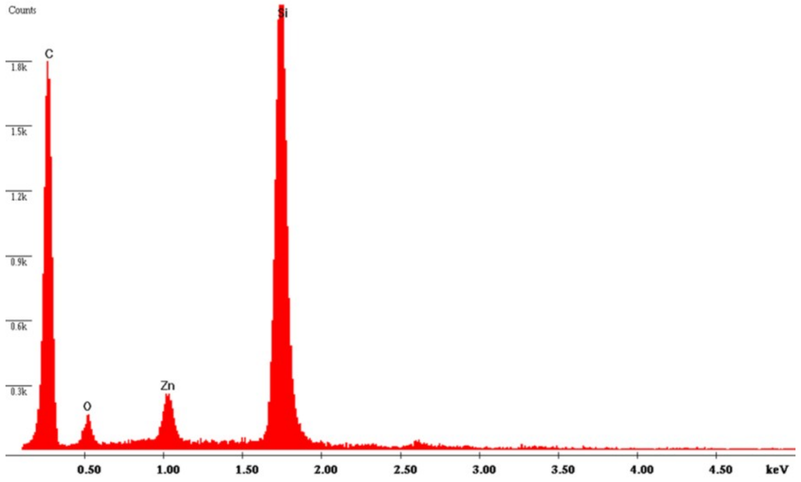

Fig. S1 EDAX analysis of nanobelts showing the presence of Zn^{II} metal ion in **NMOF-1**.

4. FT-IR Spectra

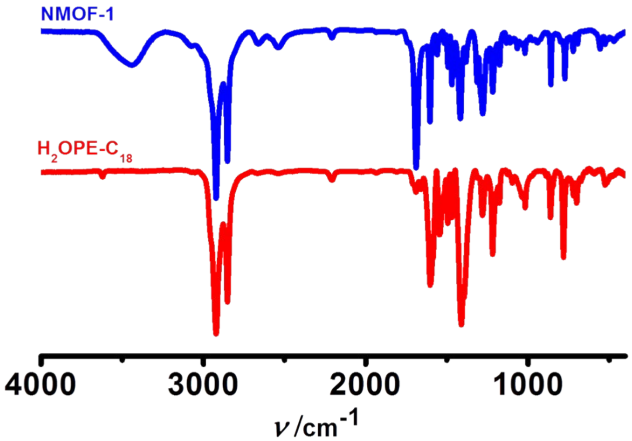

Fig. S2 FT-IR spectra of **H_2OPE-C_{18}** (red) and **NMOF-1** (blue).

5. TG Analysis

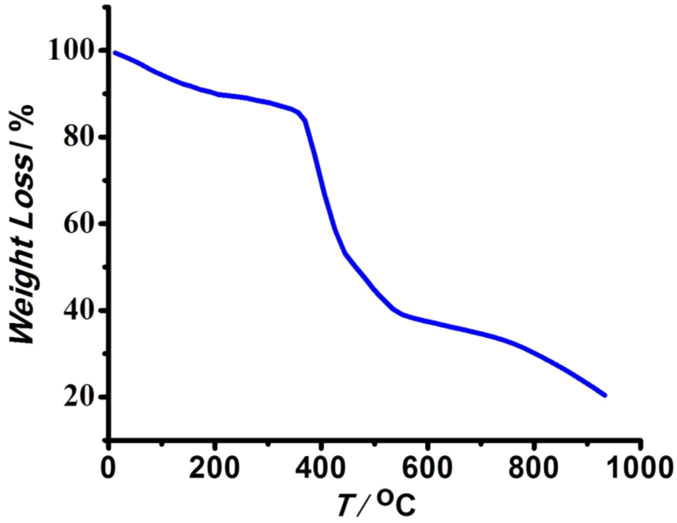

Fig. S3 TGA profile of **NMOF-1** in the temperature range 25-1000 °C with a heating rate of 3 °C/min.

6. N_2 Adsorption profile

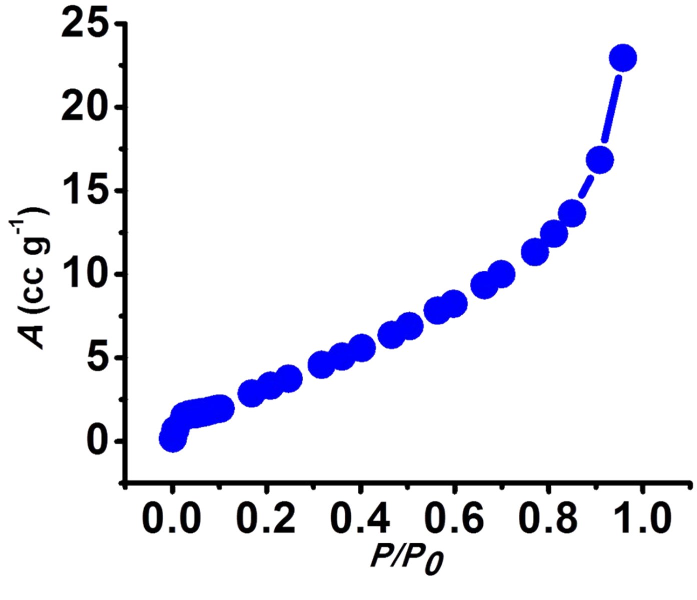

Fig. S4 N_2 adsorption isotherm of **NMOF-1** at 77 K.

7. PXRD Analysis

Table S1. Indexing data of **NMOF-1** using Crysfire software[2,3]

Cell Parameters: a = 29.40(5) Å b = 4.146(7) Å c = 22.81(5) Å β = 127.83(18) °, V = 2197 Å^3

h	k	l	D_{obs}	D_{cal}	D_{obs}-D_{cal}	Q_{obs}	Q_{cal}	$2\theta_{obs}$	$2\theta_{cal}$	*Diff*
1	0	0	23.00143	23.22249	-.22107	.00189	.00185	3.838	3.802	.037
2	0	-1	14.62921	14.69038	-.06117	.00467	.00463	6.037	6.011	.025
1	0	-2	11.01247	10.97007	.04240	.00825	.00831	8.022	8.053	-.031
3	0	-1	9.52050	9.54601	-.02551	.01103	.01097	9.282	9.257	.025
2	0	1	7.82030	7.81825	.00205	.01635	.01636	11.306	11.309	-.003
4	0	-4	5.57757	5.58686	-.00929	.03214	.03204	15.877	15.850	.027
6	0	-3	4.39640	4.38679	.00961	.04154	.04170	20.177	20.213	-.036
3	1	-1	3.80355	3.80355	.00000	.06912	.06912	23.369	23.369	.000

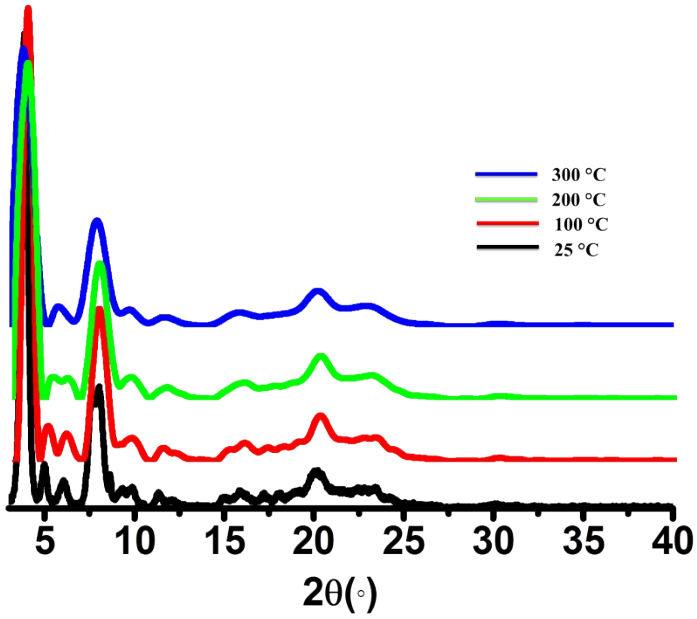

Fig. S5 Temperature dependent PXRD experiments in the temperature range 25-300 °C showing the thermal stability of **NMOF-1**

8. FESEM images of NMOF-1 nanobelts

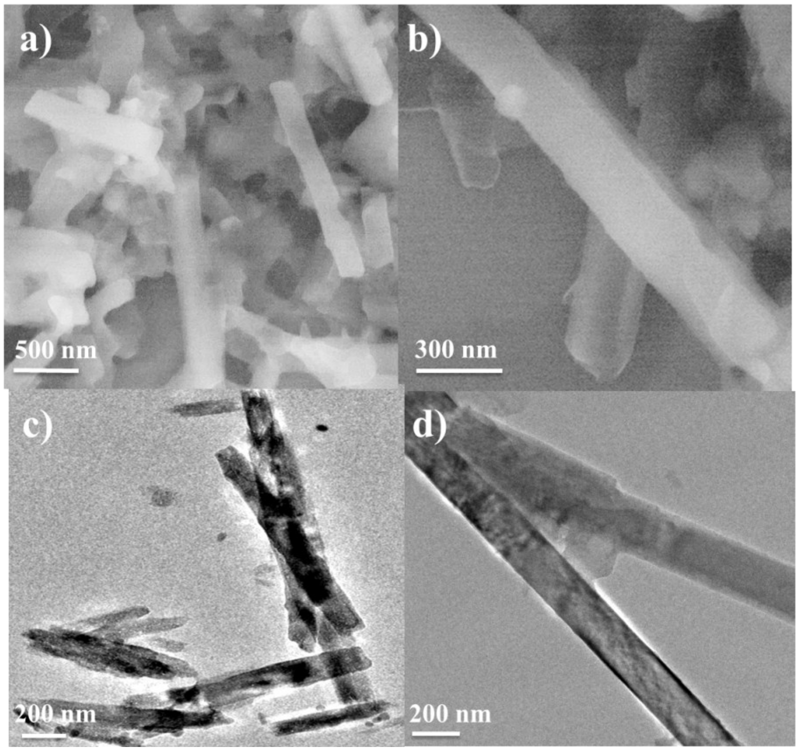

Fig. S6 (a), (b) FESEM image and (c), (d) TEM images **NMOF-1** nanobelts at different magnifications.

9. FESEM images of NMOF-1 nanoscrolls

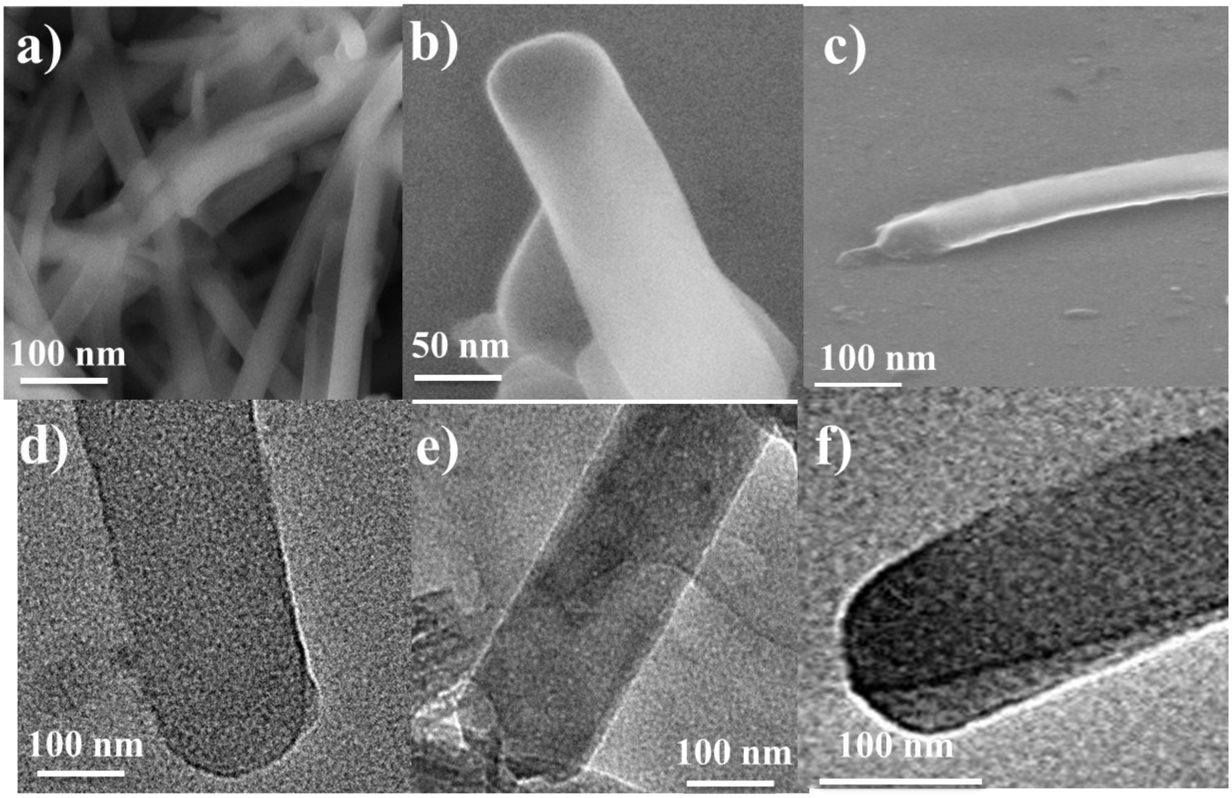

Fig. S7 FESEM image of (a) nanoscrolls, (b) and (c) nanoscrolls at higher magnifications showing changes in the cross-section on scrolling. And (d-f) TEM images of nanoscrolls showing opening at the mouth and sides.

10. FESEM images of semiscrolled NMOF-1

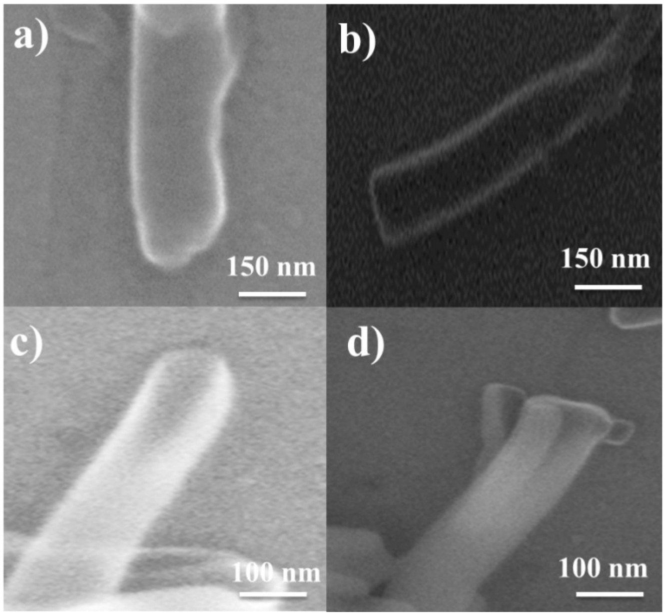

Fig. S8 FESEM images showing the morphology transformation from (a, b) nanobelt to (c, d) nanoscroll on increasing the reaction time from 2 to 6 hrs.

11. AFM Height Profile Analysis

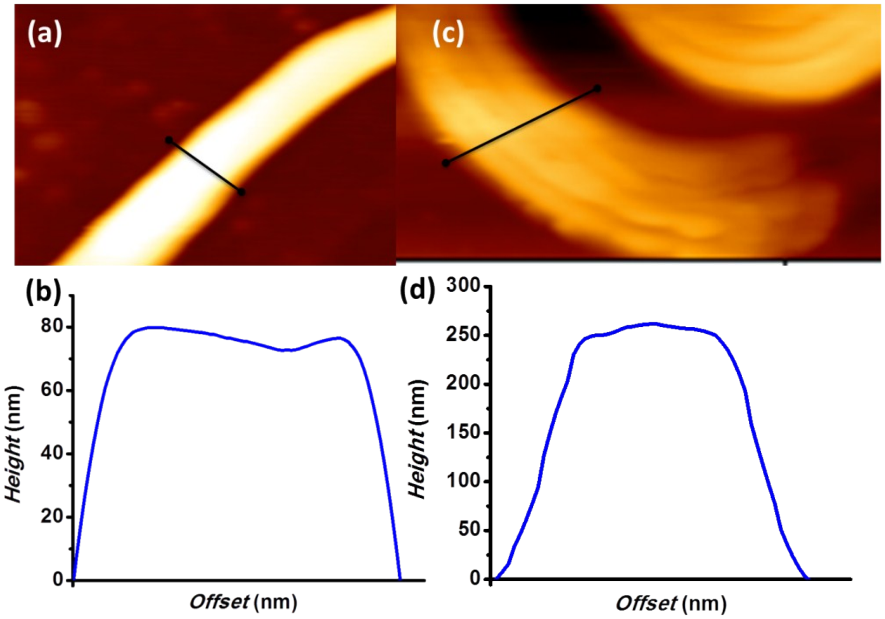

Fig. S9 AFM images and the corresponding height profile of nanobelt (a, b) and nanoscroll (c, d). The changes in height profile clearly imply the scrolling of nanobelt on increasing the reaction time from 2 to 6 hrs.

12. Structure-nanomorphology correlation

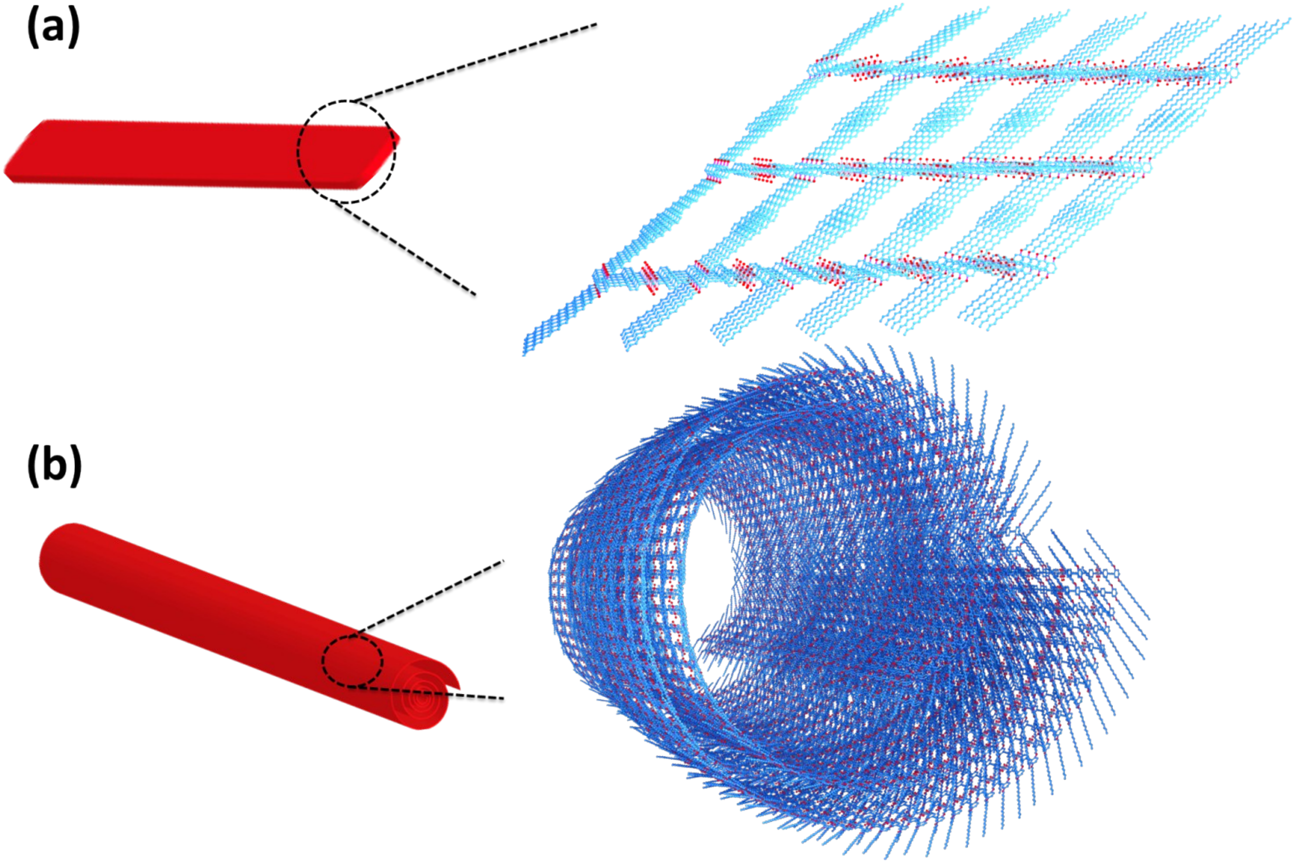

Scheme S2 Figure showing the structural correlation of **NMOF-1** (a) nanobelts from its 3D packing and (b) nanoscrolls from the rolling up of nanobelts.

13. FESEM analysis of reversibility of morphology transformation

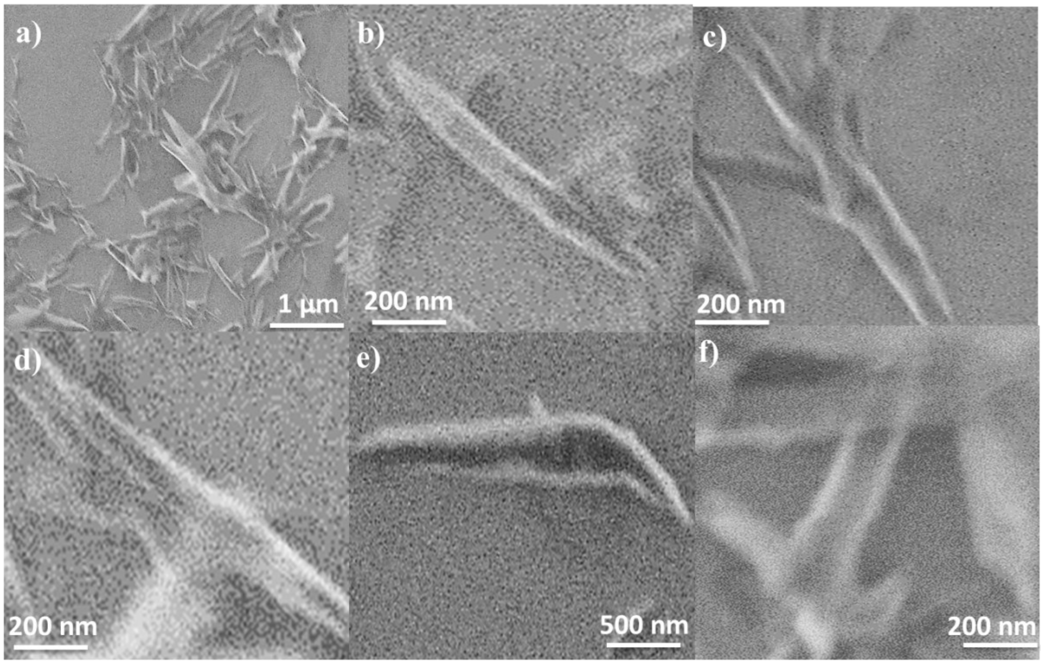

Fig. S10 (a-f) FESEM images of nanoscroll after keeping in acetonitrile solution for two days showing the opening (or unfolding) of the scrolled nanostructures.

14. Water contact angles of H_2OPEC_{18} coated glass surface

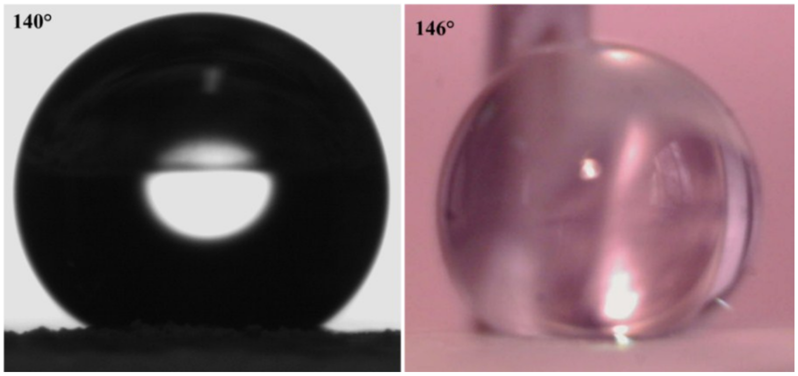

Fig. S11 Water contact angles measured on **H_2OPE-C_{18}** coated glass surface.

15. Superhydrophobicity models

Young's equation ($\cos\theta = (\gamma_{sv}-\gamma_{sl})/\ \gamma_{lv}$ where θ is the contact angle between the solid-liquid interface, $\gamma_{sv,sl\ and\ lv}$ are the corresponding surface tensions between the solid, liquid and air interfaces) is used to describe the wettability on a smooth surface. On a rough surface, the same is explained by two models: Wenzel and Cassie Baxter.[4] According to the Wenzel model, the water droplet on a rough surface is spherical in shape and wets the surface. As a consequence, it will not roll off the surface under the slightest disturbance. A transition from the Wenzel to the Cassie-Baxter model occurs when we consider that rough textures on a surface trap air-pockets in between. Therefore the water droplet cannot assume a spherical shape and rests on the rough texture with air-pockets trapped in between. This results in a low adhesion of the droplet to the surface and hence on the smallest perturbance, water rolls off the surface. It is theorized and verified experimentally that low surface energy along with a hierarchical surface roughness is essential to generate such superhydrophobic structures. A hierarchical structure implies the

presence of roughness at two regimes: micro and nano. This leads to water contact angles >150° giving rise to superhydrophobicity and self-cleaning applications.

16. FESEM analysis of NMOF-1 coated glass substrate

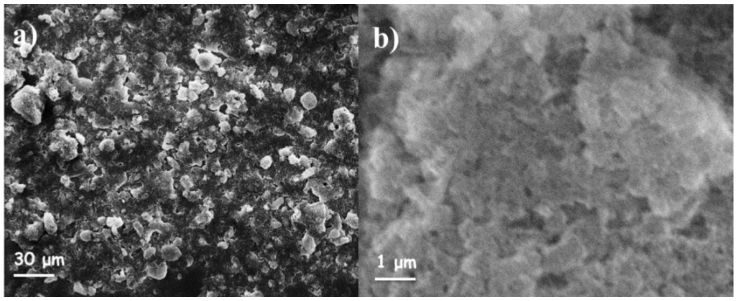

Fig. S12 (a), (b) FESEM images of **NMOF-1** coated on glass substrate at different magnifications.

17. Video Snapshots of self-cleaning experiment

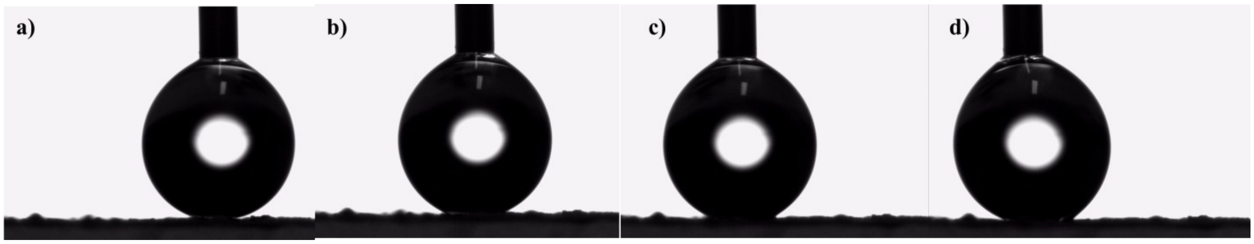

Fig. S13 (a-d) video snapshots of easy rolling of water droplet on **NMOF-1** coated surface (droplet movement from right to left).

18. Water contact angles under extreme conditions of NMOF-1 coated glass substrate

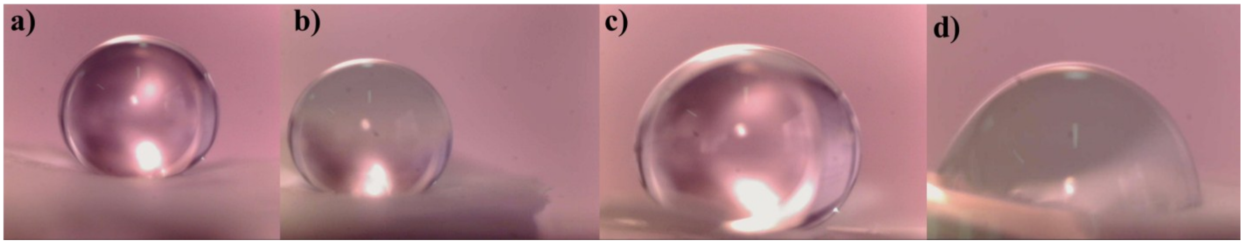

Fig. S14 Water contact angles of **NMOF-1**: (a) under acidic condition (pH of solution =1) (b) under basic condition (pH of solution = 8) (c) at high ionic strength and (d) at pH>9.

19. PXRD analysis before and after self-cleaning experiments

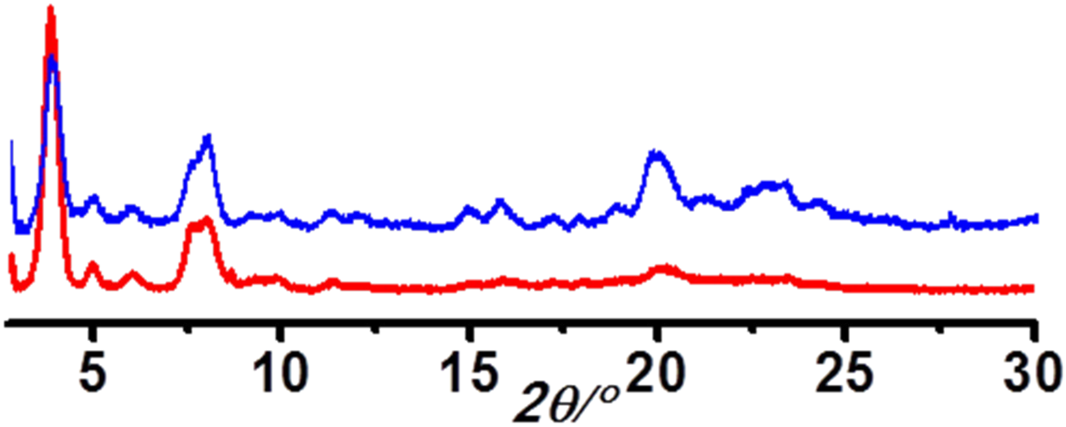

Fig. S15 PXRD pattern of **NMOF-1** before (red) and after (blue) the study of self-cleaning property with water.

20. References

1 A. R. Ramesh, K. G. Thomas, *Chem. Commun.,* 2010, **46**, 3457.

2 D. Louer, M. Louer, *J. Appl. Cryst.,* 1972, **5**, 271.

3 A. Boultif, D. Louer, *J. Appl. Cryst.,* 1991, **24**, 987.

4 X.-M. Li, D. Reinhoudt, M. Crego-Calama, *Chem. Soc. Rev.,* 2007, **36**, 1350.
